# Structural basis for RNA recognition by the C-terminal RRM domain of human RBM45

**DOI:** 10.1016/j.jbc.2024.107640

**Published:** 2024-08-08

**Authors:** Xi Chen, Qinghao Wei, Zhongmei Yang, Xiaolei Chen, Shuoxuan Guo, Meiyu Jiang, Mingzhu Wang

**Affiliations:** 1 Institutes of Physical Science and Information Technology, Anhui University, Hefei, Anhui, China; 2 School of Life Sciences, Anhui University, Hefei, Anhui, China; 3 Key Laboratory of Human Microenvironment and Precision Medicine of Anhui Higher Education Institutes, Anhui University, Hefei, Anhui, China

**Keywords:** neurodegenerative disease, amyotrophic lateral sclerosis (ALS), RNA binding protein, RNA modification, crystal structure, RNA–protein interaction

## Abstract

RBM45 is an RNA-binding protein with roles in neural development by regulating RNA splicing. Its dysfunction and aggregation are associated with neurodegenerative diseases such as amyotrophic lateral sclerosis (ALS) and frontotemporal lobar dementia (FTLD). RBM45 harbors three RRM domains that potentially bind RNA. While the recognitions of RNA by its N-terminal tandem RRM domains (RRM1 and RRM2) have been well understood, the RNA-binding property of its C-terminal RRM (RRM3) remains unclear. In this work, we identified that the RRM3 of the RBM45 sequence specifically binds RNA with a GACG sequence, similar but not identical to those recognized by the RRM1 and RRM2. Further, we determined the crystal structure of RBM45^RRM3^ in complex with a GACG sequence-containing single-stranded DNA. Our structural results, together with the RNA-binding assays of mutants at key amino acid residues, revealed the molecular mechanism by which RBM45^RRM3^ recognizes an RNA sequence. Our finding on the RNA-binding property of the individual RRM module of RBM45 provides the foundation for unraveling the RNA-binding characteristics of full-length RBM45 and for understanding the biological functions of RBM45.

RNA-binding motif protein 45 (RBM45) is an RNA-binding protein (RBP) involved in neural development (1, 2). It is highly expressed in the fetal brain, and the expression level gradually decreases with development (1). Normally, the C-terminal nuclear localization sequence (NLS) of RBM45 makes it predominantly localized to the nucleus, but RBM45 can shuttle between the nucleus and cytoplasm (3, 4). RBM45 was reported to bind to thousands of RNAs, mostly in intronic regions of pre-mRNA, regulating RNA splicing (5). Abnormalities in RBM45 have been observed to be associated with certain neurodegenerative diseases. Mutations in *RBM45* have been found linked to amyotrophic lateral sclerosis (ALS) and frontotemporal lobar dementia (FTLD) (6). In the neurons and glia cells of ALS and FTLD patients, RBM45 was found to aggregate in the cytoplasmic inclusions and co-localize with TDP-43 (TAR DNA-Binding Protein 43) (3, 7), a well-understood ALS-linked RBP (8). These results suggested that aggregation and mislocalization of RBM45 should play a role in the development of neurodegenerative diseases such as ALS and FTLD, although the mechanism remains unclear.

Characterizing the RBM45-recognizing RNAs is essential for understanding the biological function of RBM45 as well as its aggregation in cells from patients with neurodegenerative diseases. RBM45 consists of three RNA-recognition motif (RRM) domains, two at the N-terminus (RRM1 and RRM2) and one at the C-terminus (RRM3), and a pseudo-RRM domain in the middle (Fig. 1*A*). While the pseudo-RRM domain reportedly mediates self-oligomerization of RBM45, therefore named the homo-oligomer assembly (HOA) domain (3), and interacts with FUS (fused in sarcoma) (9), another well-known ALS-linked RBP (10, 11), the three RRM domains are responsible for binding RNA. Containing multiple RNA-binding modules is common in RBPs (12, 13). They recognize the same sequence or different sequences, binding RNA independently or cooperatively. Previous studies demonstrated that all three RRMs of RBM45 have RNA-binding ability (5, 14, 15). RBM45 prefers to bind RNAs containing GAC or bipartite GAC sequences *in vivo* (5). Two independent RBP recognition sequence screening studies have identified the full-length RBM45 bound GACGAC (16) or ACGC (17) sequence RNA *in vitro*. Our previous study has identified that both N-terminal RRM domains (RRM1 and RRM2) of RBM45 recognize GAC sequences (14), however, the RNA recognition property of the C-terminal RRM (RRM3) remains unclear.

**Figure 1:**
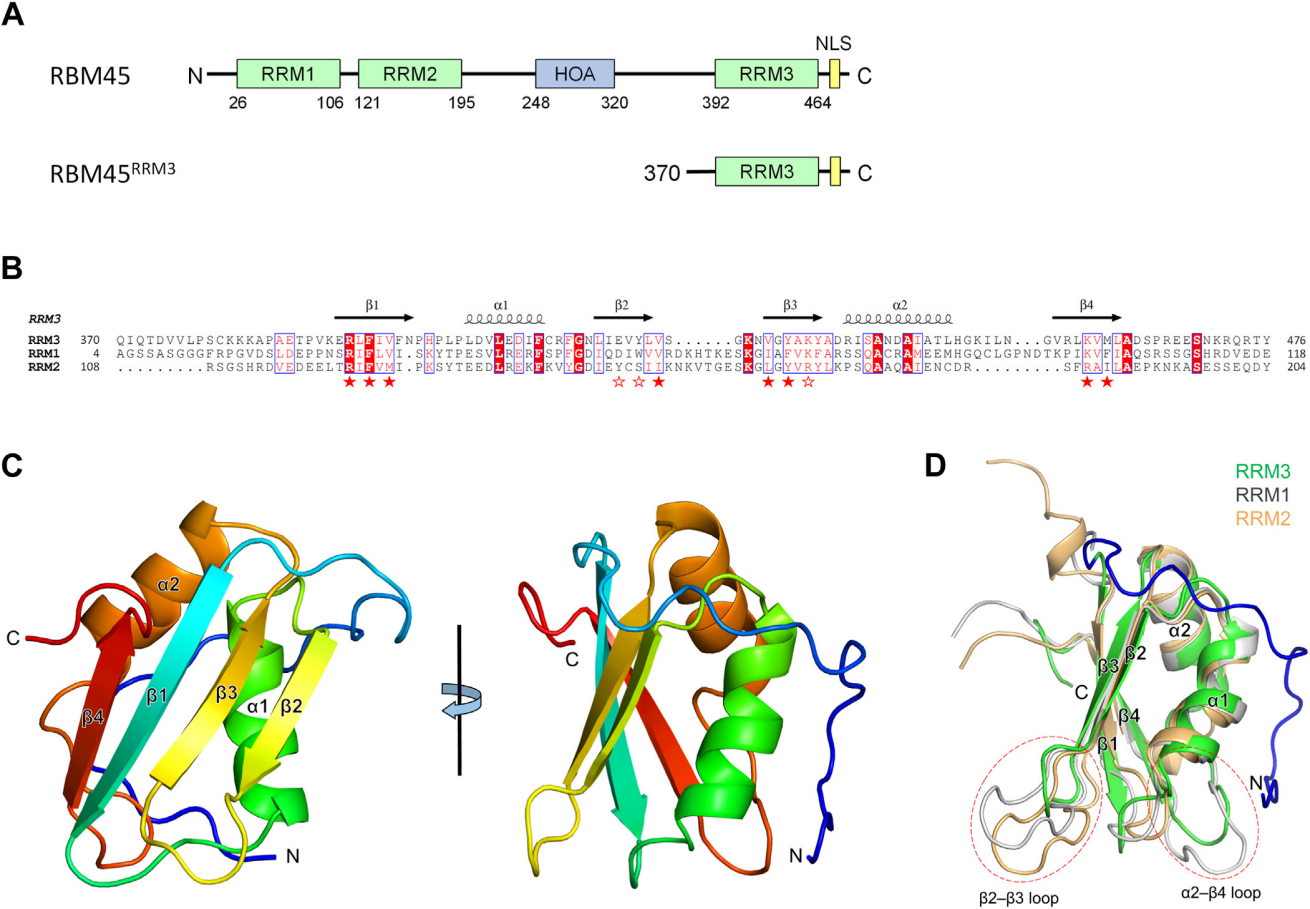
**Structure of RBM45**^**RRM3**^. *A*, the domain structure of RBM45. *B*, sequence alignment of three RRM domains of RBM45. The identical residues are highlighted in *red* background; the similar residues are highlighted in *red letters*. Residues interacting with G1, A2, and C3 through their side chains are indicated by *red pentagrams*; residues interacting with G4 through their side chains are indicated by *red blank* pentagrams. *C*, two views of the overall structure of the RBM45^RRM3^. The cartoon structure is colored in *rainbow*, with the N-terminus in *blue* and the C-terminus in *red*. *D*, structural comparison of RRM3 with RRM1 (PDB code 7CSX) and RRM2 (PDB code 7CSX) of RBM45. RRM3, RRM1, and RRM2 are colored in *green, gray*, and *wheat*, respectively. The distinct N-terminal loop of RRM3 is colored in *blue*. The β2–β3 loop and the α2–β4 loop are indicated by *red dashed circles*.

In this work, we identified that the C-terminal RRM domain of human RBM45 (RBM45^RRM3^) sequence specifically binds GACG sequence RNA and solved the crystal structures of RBM45^RRM3^ in apo form and in complex with a single-stranded DNA that mimicked the recognized RNA. Our structural result, coupled with the results of our mutation experiments, elucidated the mechanism of RNA recognition by RBM45^RRM3^.

## Results

### Crystal structure of the C-terminal RRM of RBM45

The core region (residues 392–464) of RRM3 of RBM45 shares ∼27% and ∼19% of sequence identities with RRM1 and RRM2, respectively, significantly lower than that between RRM1 and RRM2, which is about 32% (Fig. 1*B*). To determine the structure of the C-terminal RRM (RRM3) of RBM45, we tried a series of different RBM45 fragments containing RRM3 for crystallization, and finally obtained crystals of a fragment from residue 370 to the C-terminus (hereafter named RBM45^RRM3^, Fig. 1*A*) and resolved its crystal structure at 2.4-Å resolution. There are four RBM45^RRM3^ molecules per asymmetry unit, containing residues from Gln370 to Arg466 or Glu467. The last nine residues at the C-terminus, including the NLS, are disordered in all four molecules. Except for a few amino acid residues at the very N-terminus (residues 370–373), there is no significant difference between the four chains (Fig. S1), so only one chain will be described hereafter. RBM45^RRM3^ bears the canonical RRM fold, which is characterized by a βαββαβ topology. The four β-strands form an antiparallel β–sheet in the order of β4–β1–β3–β2, while the two α-helices are located on the same side of the sheet (Fig. 1*C*). The exposed side of the β-sheet is predominantly positively charged, indicating a potential RNA binding surface (Fig. S2). The overall structure of RBM45^RRM3^ can be well aligned with the AlphaFold-predicted model (18) with a Cα root-mean-square deviation (RMSD) of 0.42 Å (for 86 residues). The major differences occur in the N-terminal loop, C-terminal loop, and some other loop regions (Fig. S3), and these regions also show diversity among the four molecules in the asymmetric unit (Fig. S1).

The overall fold of RBM45^RRM3^ is similar to RRM1 and RRM2 of RBM45 (PDB code 7CSX), with Cα RMSDs of 0.86 Å (for 61 residues) and 1.06 Å (for 46 residues), respectively. However, they are significantly different in some loop regions. The β2–β3 loop of RRM3 is much shorter than those of RRM1 and RRM2, whereas the α2–β4 loop of RRM3 is a little shorter than that of RRM1 and significantly longer than that of RRM2. The most significant difference is that RRM3 has a distinctive N-terminal loop surrounding α1 and touching α2 as well as the α2–β4 loop (Fig. 1*D*). However, this pre-RRM loop, which is absent in RRM1 and RRM2, as well as in other structurally known RRMs, is not involved in the predicted RNA binding surface (Figs. 1*D* and S2).

### The C-terminal RRM of RBM45 recognizes GACG RNA

**Figure 2:**
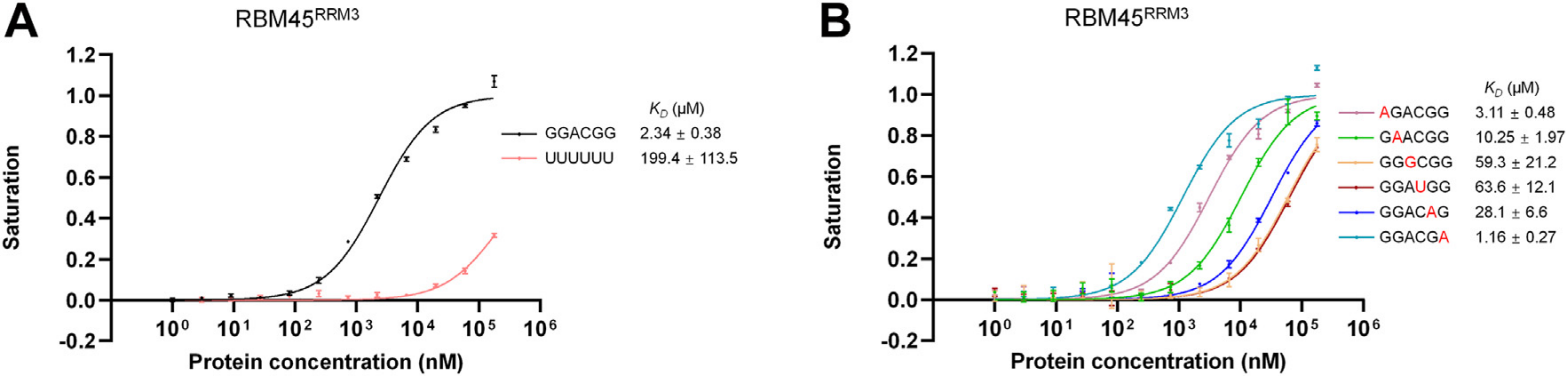
**The RNA-binding property of RBM45**^**RRM3**^. *A*, the fluorescence polarization (FP) measurements of the binding affinities of RBM45^RRM3^ to the GGACGG sequence and polyU 6-mer RNA. *B*, the FP measurements of the binding affinities of RBM45^RRM3^ for RNA 6-mers substituted at each site. For brevity, the results of only one representative substitution for each site are shown here, and the full FP results of substitutions are shown in Fig. S5. The substituted bases are highlighted in *red*. The data shown here are the averages of three replicates. The error bars indicate the standard deviations of three replicates.

Previous studies have shown that both the RRM1 and RRM2 domains of RBM45 recognized GAC sequence RNA (14). To identify whether RBM45^RRM3^ recognizes the same sequence, we analyzed the binding of RBM45^RRM3^ with GAC-contain RNA using fluorescence polarization (FP) assay. RBM45^RRM3^ bound a 5′-FAM labeled 6-mer RNA, 5′-GGACGG-3′, with a much higher affinity (dissociation constant (*K*_*D*_) of 2.34 ± 0.38 μM) than that for a non-specific sequence (polyU) 6-mer RNA (with *K*_*D*_ of 199.4 ± 113.5 μM) (Fig. 2*A*), suggesting that RBM45^RRM3^ binds RNA sequence-specifically and likely also prefers the GAC sequence. We also analyzed the binding affinity of RBM45^RRM3^ to single-stranded DNA (ssDNA). The FP results showed that RBM45^RRM3^ bound 5′-GGACGG-3′ ssDNA 6-mer with a *K*_*D*_ of 8.77 ± 0.61 μM (Fig. S4), suggesting that RBM45^RRM3^ can also bind ssDNA, although the affinity is much weaker than that for RNA.

To identify the precise recognition sequence of RBM45^RRM3^, we substituted each residue of the 6-mer RNA with other nucleotides and analyzed the changes in binding affinities using the FP method. The substitutions of the second guanine, the adenine, the cytosine, or the third guanine of the 6-mer RNA resulted in ∼4-fold, 25–30-fold, 26–39-fold, or 6.5–14-fold reductions of the RBM45^RRM3^-binding affinity, respectively, whereas the substitutions in the first and last guanines did not significantly change the RBM45^RRM3^-binding affinity (Figs. 2*B* and S5). It should be noted that since the assays for some RNA variants failed to reach saturation of binding, the calculation of *K*_*D*_ values for these assays should be considerably inaccurate. Nevertheless, the FP results still clearly showed that these sequence variants significantly reduced the RRM45^RRM3^-binding affinity of RNA. These results demonstrated that RBM45^RRM3^ recognizes the GACG sequence, which is slightly different from the GAC sequence recognized by RRM1 and RRM2 of RBM45.

As RBM45^RRM3^ was recently reported as an N6-methyladenosine (m^6^A) reader, we also analyzed its binding ability for m^6^A-containing RNA. The FP result showed that RBM45^RRM3^ bound 5′-FAM labeled GG(m^6^A)CGG 6-mer RNA with a *K*_*D*_ of 4.62 ± 0.55 μM (Fig. S6), slightly weaker than that for unmethylated RNA, which implied that RBM45^RRM3^ does not exhibit specific affinity for m^6^A modified RNA.

### Crystal structure of RBM45^RRM3^ in complex with ssDNA

**Figure 3:**
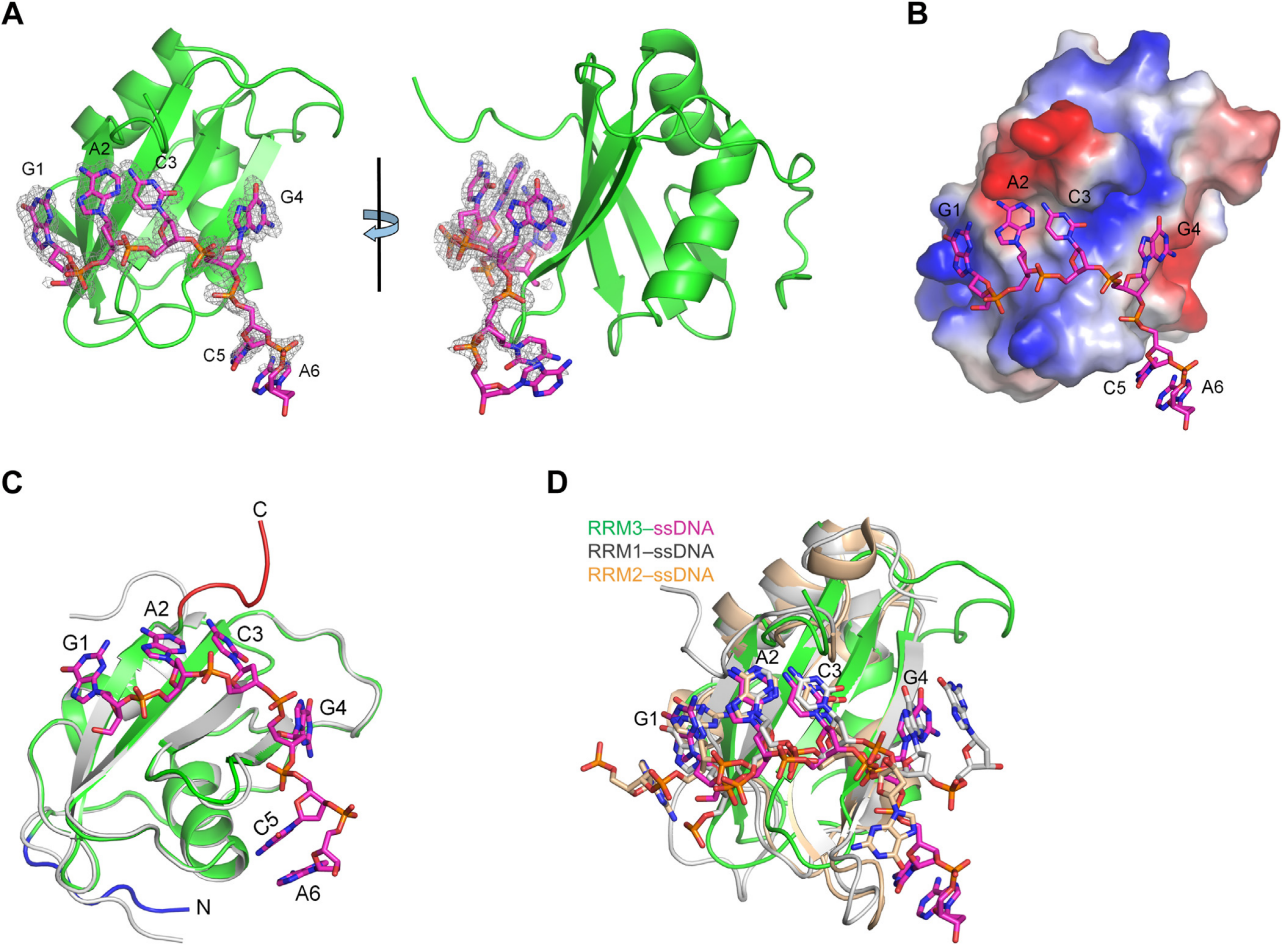
**Structure of RBM45**^**RRM3**^**in complex with ssDNA**. *A*, two views of the overall structure of RBM45^RRM3^–ssDNA complex. RBM45^RRM3^ is presented as a *green cartoon*; ssDNA is shown as *magenta sticks*. The simulated annealing map of ssDNA (F_o_–F_c_ map contoured at 3.0 σ) is shown as *gray meshes*. *B*, the surface electrostatic potential of RBM45^RRM3^. Positively charged and negatively charged areas are indicated in *blue* and *red*, respectively. ssDNA is presented as *magenta sticks*. *C*, structural comparison of RBM45^RRM3^ in the complex and protein-alone structures. The complex structure and the protein-alone structure are presented as *green* and *gray cartoons*, respectively. The N-terminus and C-terminus of RBM45^RRM3^ in the complex structure are highlighted in *blue* and *red*, respectively. *D*, comparison of RBM45^RRM3^–ssDNA structure with RRM1–ssDNA and RRM2–ssDNA structures (PDB code 7CSZ). RRM3, RRM1, and RRM2 are presented as *green, gray*, and *wheat cartoons*, respectively; the RRM3-, RRM1-, and RRM2-bound ssDNA are presented as *magenta, gray*, and *wheat sticks*, respectively.

To explore the RNA-recognition mechanism of RBM45^RRM3^, we attempted to co-crystallize RBM45^RRM3^ with RNA or ssDNA containing GACG sequence and finally solved the crystal structure of RBM45^RRM3^ in complex with an ssDNA 7-mer, 5′-GACGCAG-3′, at a resolution of 1.65 Å. There are also four RBM45^RRM3^ molecules per asymmetric unit, however, the space group and the packing pattern of the complex crystal are totally different from those of the protein-alone crystal. Clear electron densities of ssDNA can be observed at the presumed RNA binding site, allowing unambiguous ssDNA model-building (Fig. 3, *A* and *B*). The final model includes the first six nucleotides of the ssDNA 7-mer, hereafter named from G1 to A6. The structures of the four protein chains, as well as their bound ssDNAs, are very similar (Fig. S7), thus we will describe only one RBM45^RRM3^ chain and its bound ssDNA hereafter. The ssDNA binds to the positively charged β-sheet surface of RBM45 with its 5′ end bound to the β4 side and its 3′ end bound to the β2 side of the β-sheet, consistent with the conventional RNA binding pattern of RRMs (19). There was no direct interaction observed between the pre-RRM loop and ssDNA (Fig. 3*A*). Superimposition with the protein-alone structure shows that the C-terminal loop of RBM45^RRM3^ in the complex structure is in a different direction (Fig. 3*C*). This loop is directly involved in the interaction with ssDNA, suggesting that the conformational change is most likely a result of ssDNA binding. The ssDNA/RNA-binding-induced conformational changes of the C-terminal loop have also been found in other RRMs, such as RRM1 and RRM2 of RBM45 (14). The most N-terminus of RBM45^RRM3^ also exhibits different conformations in the two structures (Fig. 3*C*). However, this region does not interact with the ssDNA, and it also shows conformational variety in the protein-alone structure (Fig. S1), suggesting that this conformational inconsistency is more likely due to crystal packing. The Cα RMSD between the RBM45^RRM3^ structure in the complex and the AlphaFold-predicted model is about 0.30 Å (for 74 residues), comparable to that of the protein-alone structure. Interestingly, in those regions that directly interact with ssDNA, such as the C-terminal loop and the β2–β3 loop, the AlphaFold-predicted model is more similar to the complex structure, whereas in regions that do not contact ssDNA, such as the most N-terminal loop, the AlphaFold-predicted model is more similar to the protein-alone structure (Fig. S8).

Compared with the structure of the RBM45^RRM1-2^–ssDNA complex (PDB code 7CSZ), which also contained the GACG motif, the binding patterns of A2 and C3 are remarkably consistent with the corresponding bases bound by RRM1 and RRM2. Three RRMs of RBM45 bind G1 (or the corresponding guanine) at similar sites, but the orientations of the purine rings of the three guanines are different. The purine rings of guanines bound by RRM3 or RRM1 are approximately parallel to the β-sheet, whereas the purine ring of RRM2-bound guanine is almost perpendicular to the β-sheet. The corresponding guanines of G4 bind at quite different positions in RRM1 and RRM2, and the binding of G4 in RRM3 is closer to that in RRM1 (Fig. 3*D*).

### Structural details of nucleic acids recognition

**Figure 4:**
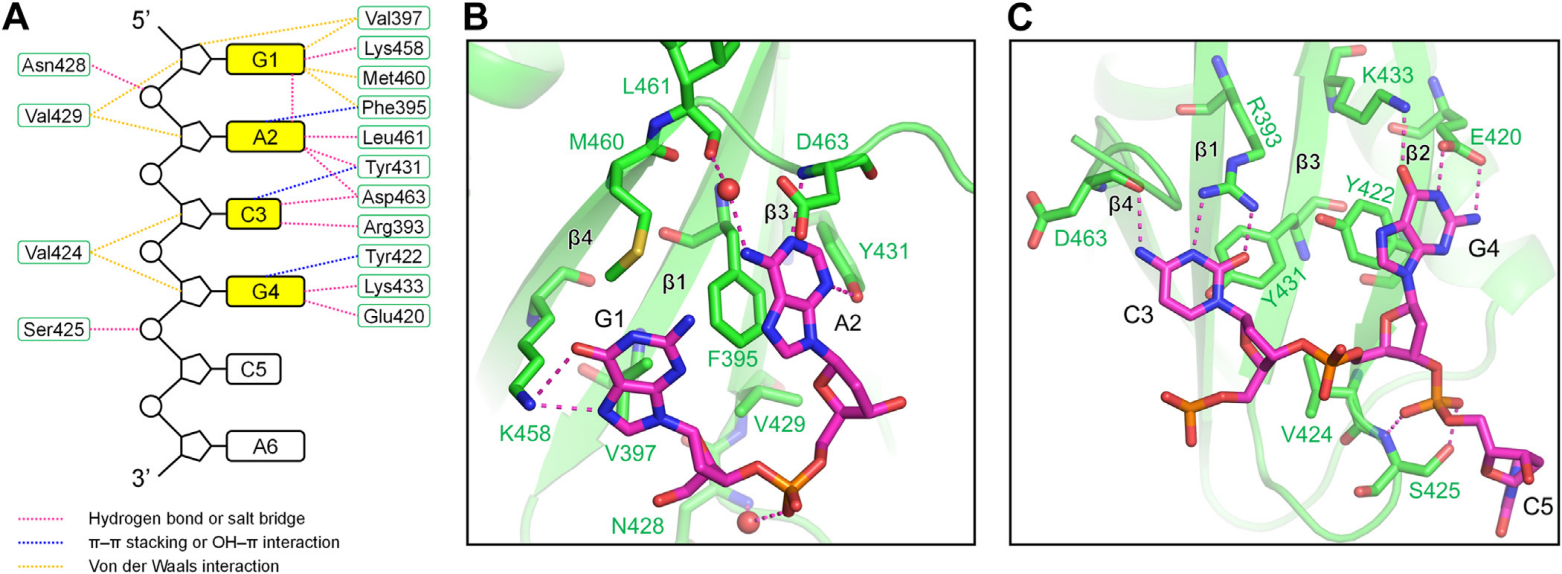
**Structural details of nucleic acids recognition**. *A*, schematic diagram of RRM45^RRM3^-ssDNA interaction. The bases, deoxyriboses, and phosphates are represented as *rectangles, pentagons*, and *circles*, respectively. The recognized bases are highlighted in *yellow* background. *B*, interactions of RBM45^RRM3^ with G1 and A2. *C*, interactions of RBM45^RRM3^ with C3 and G4. Protein chains are presented as *green cartoons*; nucleotide residues are presented as *magenta sticks*; amino acid residues that interacted with ssDNA are presented as *green sticks*; hydrogen bonds are indicated by *magenta dashed lines*.

The structure of RBM45^RRM3^–ssDNA complex presents the details of how RRM3 of RBM45 recognizes each base of ssDNA. Specifically, G1 binds to a hydrophobic patch formed by the side chains of Phe395 and Val397 in β1 and Met460 in β4; a γ-methyl of Val397 side chain contacts the purine ring of G1 *via* the CH–π interaction; the side-chain amino group of Lys458 in β4 forms hydrogen bonds with N7 and O6 of G1 (Fig. 4, *A* and *B*). The purine ring of A2 π–π stacks with the aromatic side chain of Phe395; the N1 and N3 of A2 form hydrogen bonds with the main-chain amino group of Asp463 in the C-terminal loop and the side-chain hydroxyl group of Tyr431 in β3, respectively; the N6 forms a hydrogen bond with the main-chain carbonyl group of Leu461 *via* a water molecule (Fig. 4, *A* and *B*). The hydroxyl group of Tyr431 contacts the pyrimidine ring of C3 through OH–π interaction; two side-chain amino groups of Arg393 in β1 form hydrogen bonds with O2 and N3 of C3; the main-chain carbonyl group of Asp463 forms a hydrogen bond with N4 of C3. The aromatic side chain of Tyr431 does not stack with C3 but instead stacks with the guanidine group of Arg393, stabilizing its conformation (Fig. 4, *A* and *C*). For G4, its purine ring π–π stacks with the side chain of Tyr422, whereas its N1 and N2 form two hydrogen bonds with the side-chain carboxyl group of Glu420 in β2 and its O6 forms a hydrogen bond with the side-chain amino group of Lys433 in β3. In addition to interactions with the base moieties of ssDNA, RBM45^RRM3^ also contacts the backbone of ssDNA. The ribose moieties of G1 to G4 bind to a hydrophobic patch formed by side chains of Val429, Phe395, and Val424, whereas the phosphate of A2 forms hydrogen bonds with the main-chain amino of N428 *via* a water molecule (Fig. 4, *A*–*C*). The densities of C5 and A6 are weak (Fig. 3*A*), suggesting that the structures of C5 and A6 are largely the result of crystal packing. C5 interacts weakly with RBM45^RRM3^, except that the phosphate of C5 forms two hydrogen bonds with the main-chain amino group and the side-chain hydroxyl group of Ser425 (Fig. 4, *A* and *C*), whereas A6 does not directly contact the protein (Fig. 3, *A* and *B*). It should be noted that introducing 2′-hydroxyl groups to ssDNA in the complex structure does not result in any observable steric hindrance, which suggests that our complex structure might also represent the binding of RNA. Furthermore, the fact that RBM45^RRM3^ binds RNA with significantly stronger affinity than DNA suggests that the 2′-hydroxyl groups may introduce additional interactions, although these interactions are hard to extrapolate from our protein-ssDNA complex structure. Interestingly, the side chains of several key amino acids, including Phe395, Arg393, and Tyr431, exhibited distinct conformations in the complex structure compared with those in the protein-alone structure (Fig. S9), implying that the side chains of these residues may undergo conformational changes during RBM45^RRM3^ bind RNA.

### Binding assays of the interaction surface

**Figure 5:**
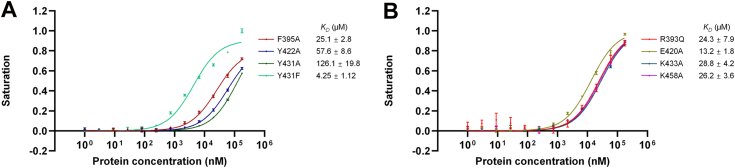
**RNA-binding affinities of RBM45**^**RRM3**^**mutants**. *A*, FP measurements of RNA-binding affinities of three key aromatic amino acid mutants. *B*, FP measurements of RNA-binding affinities of several other key residue mutants. The data shown here are the averages of three replicates. The error bars indicate the standard deviations of three replicates.

To validate the ssDNA/RNA-binding mechanism revealed by our structural results, we generated single mutants of several key amino acid residues and measured their RNA-binding affinities using the FP method. Single mutations of two aromatic residues, Phe395 and Tyr422, which stack with the purine rings of A2 and G4, respectively, to alanine reduced the RNA-binding affinity by ∼10- and ∼25-fold, respectively (Fig. 5*A*), suggesting that they both play important roles in RNA-binding. Mutating Tyr431 to phenylalanine or alanine reduced the RNA-binding affinity by 1.8- or ∼50-fold, respectively (Fig. 5*A*), suggesting that in addition to the direct interaction of its side-chain hydroxyl group with the adenine and cytosine bases, the stabilization of Arg393 side-chain conformation may be more important for RNA binding. To verify the importance of Arg393, which forms two hydrogen bonds with the base of cytosine, in RNA binding, we attempted to generate the R393A mutant, but it was not expressed well in *E. coli*. However, we successfully obtained the R393Q mutant protein, which showed an approximately 10-fold decrease in RNA-binding affinity, proving that Arg393 is critical for RNA binding (Fig. 5*B*). For Lys458, which forms hydrogen bonds with the first guanine of the GACG sequence, the K458A mutation reduced RNA-binding affinity by ∼11-fold. For Glu420 and Lys433, which form hydrogen bonds with the last guanine of the GACG sequence, the E420A and K433A mutations reduced the RNA-binding affinities by ∼5- and 12-fold, respectively (Fig. 5*B*). These results indicate that these amino acid residues that form hydrogen bonds with bases also play crucial roles in RNA binding. We would like to caution that the binding assays for some mutants did not reach saturation, therefore, the *K*_*D*_ calculations for these assays might be inaccurate. However, our FP results still clearly showed that these mutations significantly reduced the RNA-binding affinity of RBM45^RRM3^.

## Discussion

In this study, we verified that the RRM3 domain of RBM45 binds RNA in a sequence-specific manner like its RRM1 and RRM2 domains, and identified that RBM45^RRM3^ recognizes RNA sequence GACG, which is slightly different from the GAC sequence recognized by RRM1 and RRM2. Our structural and biochemical results showed that three aromatic residues, Phe395 in β1, Tyr431 in β3, and Tyr422 in β2, play crucial roles in RNA binding. Two of them, Phe395 and Tyr422, stack with A2 and G4, respectively, whereas Tyr431 does not stack with any base but contacts the pyrimidine ring of C3 through OH–π interaction and stacks with Arg393, another key amino acid residue for RNA binding. Stackings of these aromatic side chains with the bases provide the essential driving force to bind RNA. Single mutations in these aromatic residues significantly weakened the RNA-binding affinity. Several other amino acid residues form hydrogen bonds with bases through their side-chain or main-chain groups, further promoting the binding to RNA and supposedly providing sequence-specificity (20). Among them, for those residues that interact with bases through side chains, including Arg393, Glu420, Lys433, and Lys458, their single mutations also significantly reduced the RNA-binding affinities. These residues, as well as the three aromatic residues described above, are conserved in RBM45s from *Drosophila* to humans (Fig. S10), suggesting that the mechanism of RNA recognition by RBM45^RRM3^ should be conserved across species. Mutation in one of these residues, Lys458, has been found linked to ALS and FTLD (6), suggesting that the RNA-binding ability/specificity of RBM45 is likely to play an important role in the development of these neurodegenerative diseases.

Our biochemical results showed that RBM45^RRM3^ exhibited different specificities for different nucleotides among the recognized GACG sequences. RBM45^RRM3^ is highly selective for the central dinucleotide of the GACG sequence, adenine and cytosine, as substituting any of them with any other nucleotides resulted in a more than 25-fold decrease in affinity. In contrast, substituting the first guanine in the GACG sequence with other nucleotides only reduces the affinity by ∼4-fold. RBM45^RRM3^ is moderately selective for the latter guanine, with a 6.5–14-fold higher affinity for guanine than for other nucleotides. Our structural result revealed that the adenine and cytosine are bound to RBM45^RRM3^ through interactions between their bases and the conserved aromatic residues in RNP2 and RNP1, respectively, which is the classical mechanism for nucleotide binding by RRM domains (19). In addition, two bases each form three hydrogen bonds with the main-chain or side-chain atoms of RBM45^RRM3^. The base of the last guanine stacks with the aromatic side chain of Tyr422, a non-classical binding site in β2, and forms three hydrogen bonds with side-chain atoms of RBM45^RRM3^. In contrast, the first guanine base interacts less with the protein; it does not stack with any aromatic residues and only forms two hydrogen bonds with the protein through the side chain amino group of Lys458.

**Figure 6:**
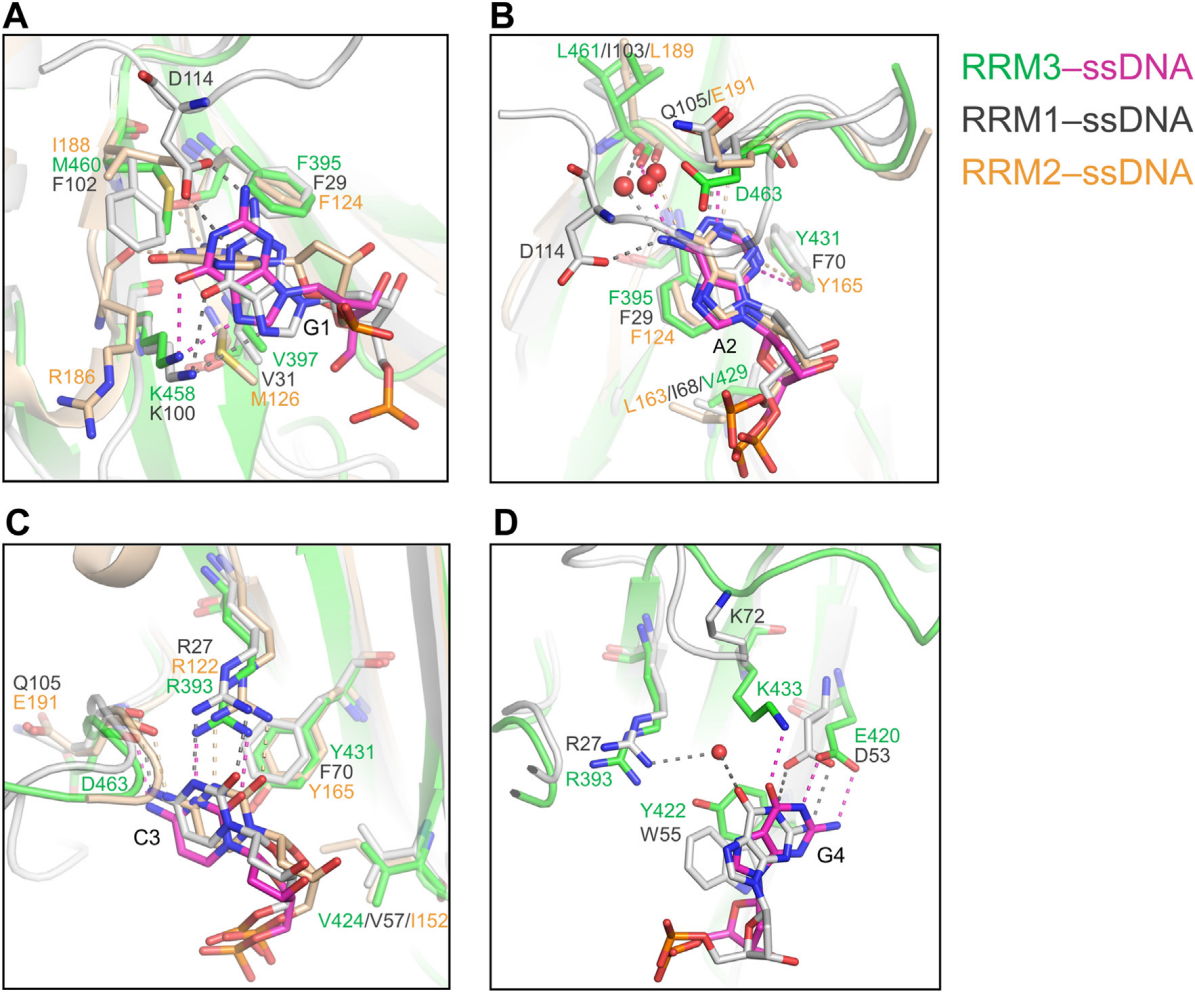
**Structural comparison of the ssDNA binding detail of three RRMs of RBM45**. *A–C*, the structural comparisons of G1, A2, and C3 binding of three RRMs, respectively. *D*, the structural comparison of G4 binding of RRM3 and RRM1. The key amino residues in RRM3, RRM1 (PDB code 7CSZ), and RRM2 (PDB code 7CSZ) are presented as *green, gray*, and *wheat sticks*, respectively; the RRM3-, RRM1-, and RRM2-bound ssDNA are presented as *magenta, gray*, and *wheat sticks*, respectively; the hydrogen bonds in RRM3–ssDNA, RRM1–ssDNA, and RRM3–ssDNA complexes are indicated by *magenta, gray*, and *wheat dashed lines*, respectively.

RBM45^RRM3^ shares a portion of its recognition sequence, GAC, with RRM1 and RRM2. However, the mechanisms of the three RRMs recognizing these three nucleotides are not exactly the same. The recognition of the core dinucleotide, the adenine and the cytosine, are conserved among the three RRMs of RBM45. The two nucleotides recognized by RBM45^RRM3^ can be superimposed well with those bound by RRM1 and RRM2. The purine rings of adenines all stack with the conserved phenylalanine in RNP2, and each purine ring forms two hydrogen bonds with main-chain groups in the C-terminal loop. A minor difference is that the bases of RRM3-and RRM2-bound adenine each form an additional hydrogen bond with the side-chain hydroxyl group of the conserved tyrosine in RNP1, whereas there is no such hydrogen bond in RRM1-bound adenine, as the corresponding aromatic residue in RRM1 is phenylalanine (Figs. 1*B* and 6*B*). For the cytosine, the pyrimidine rings all form two hydrogen bonds with the side chain of the arginine (Arg393/Arg27/Arg122) in RNP2 and one hydrogen bond with a main-chain carbonyl group in the C-terminal loops, and all interact with the side chain of the conserved aromatic residues (Y431/F70/Y165) in RNP1 (Fig. 6*C*). In contrast, the three RRMs interact with the first guanine quite differently. Comparatively, the binding of RRM3 is more similar to that of RRM1. However, the base of the RRM1-bound guanine forms two additional hydrogen bonds with the side-chain carboxyl group of Asp114 in the C-terminal loop. The purine ring of RRM2-bound guanine exhibits a different orientation. It is sandwiched between the side chains of Met126 and Ile188 and forms two hydrogen bonds with the main-chain carbonyls of Phe124 and Arg186 (Fig. 6*A*). The observed interaction of RRM3 with the base of this guanine is less than that of either RRM1 or RRM2, consistent with the fact that RRM3 is relatively less selective for the first guanine of its recognized sequence. Although RRM1 does not sequence-specifically recognize the last guanine of GACG as RRM3 does, binding of this guanine is also observed in the RRM1–ssDNA structure. Interestingly, the details of the two RRMs binding this guanine are similar. The RRM1-bound guanine also stacks with the side chain of an aromatic residue (Trp55) in α2 and forms two hydrogen bonds with an acidic residue (Asp53) in α2 as the RRM3-bound guanine does. The RRM3-bound guanine forms an additional hydrogen bond with the side chain of a lysine residue (Lys433) in α3, whereas the RRM1-bound guanine interacts with the side chain of an arginine residue (Arg27) in α1 through a water molecule (Fig. 6*D*). However, although RRM1 binds this guanine in a manner similar to that of RRM3, biochemical assays showed that its binding to this guanine is sequence nonspecific (14). One possible explanation might be that stacking with tryptophan and hydrogen bonding mediated by water may confer more flexibility to the binding site of RRM1, thus allowing it to bind other nucleotides, whereas this binding site in RRM3 is less flexible and only suitable for binding guanine.

RBM45 is reportedly an m^6^A reader that recognizes m^6^A through its RRM3 domain (5). However, our binding assays showed that RBM45^RRM3^ did not preferentially target m^6^A. Our structural data also did not support that RBM45^RRM3^ prefers to recognize m^6^A. Our complex structure indicated that the N6 methyl group of m^6^A in the *syn* conformation, which is the major conformation of m^6^A (21, 22), neither introduces additional interactions nor leads to steric hindrance (Fig. S11), suggesting that RBM45^RRM3^ is unlikely to be selective for the N6 methylation of adenine. There are two possible reasons for the inconsistency of our results with the previously reported results: one is that the HOA domain, which is not included in our biochemical and structural studies, may affect the binding of RRM3 to m^6^A; the other is that N6 methylation of adenine may destabilize the Watson-Crick base pairing, affecting the secondary structure of RNA and predisposing the N6 methylated GACG motifs to adopt single-stranded conformation (23, 24), which would be more favorable for RBM45 binding. Either reason would make full-length RBM45 prefer to bind N6-methylated RNAs *in vivo*, although a single RRM domain does not exhibit any preference for N6-methylation modification of short RNA fragments *in vitro*.

RBM45 contains three RRM domains, which all have the ability to bind RNA. Their recognition sequences are similar, but not identical. Containing multiple RRM domains is common in RBPs. The RNA binding specificity and affinity of individual RRM domains are usually limited, and the coordination of multiple RRMs will help to improve the RNA-binding specificity and/or affinity (12, 25). Although for most multiple-RRM RBPs, the RNA sequences recognized by their individual RRM domains are not well characterized to date, available studies with clear biochemical and/or structural evidence have shown that different RRMs of the same RBP may either recognize similar sequences or very different sequences. For example, all three RRMs of HuR bind polyU sequences (26, 27, 28), and its RRM3 can also bind UUA (28); the RRM1, RRM3, and RRM4 of PTB all recognize UCU, while its RRM2 recognizes CU (29); RRM1 and RRM2 of TDP-43 recognize GGUG and UG, respectively (30); and RRM1 and RRM2 of hnRNP A1 recognize UAG(G) and (U)AG (the sequences in parentheses denote the variations in the results of different studies), respectively (31, 32, 33). The recognition sequences of the different RRMs in these RBPs are identical or similar and share a core sequence, UU for HuR, CU for PTB, UG for TDP-43, and (U)AG for hnRNP A1. This is also the case for RBM45; although the sequences recognized by the three RRMs are not identical, they share a core trinucleotide, GAC. In contrast, for some RBPs, the sequences recognized by their different RRMs are quite different. For example, RRM1 of RBM5 recognizes GG (34), while its RRM2 binds CU or GA (35); RRM2 and RRM3 of Prp24 recognize (G)AG(A) and AAC, respectively, while its RRM1 and RRM4 do not bind RNA (36, 37, 38). While the N-terminal tandem RRM domains (RRM1 and RRM2) interact with each other to form two antiparallel RNA-binding sites both binding GAC sequences (14), the C-terminal RRM domain (RRM3) is predicted not to interact tightly with any other domains (18), resulting in a separate binding site recognizing GACG sequence. The 3-nucleotide or 4-nucleotide recognition sequence implies a limited sequence specificity. Whereas multiple RRM domains with contiguous RNA-binding sites improve both the sequence-specificity and affinity of RBPs, multiple RRM domains that do not form contiguous binding sites, as in the case of RBM45, can only improve the RNA-binding affinity and capacity (19, 20, 39). RBM45 most likely prefers to bind RNAs containing multiple GAC or GACG motifs. RBM45 was identified to recognize GAC or bipartite GAC sequences RNAs *in vivo* (5), consistent with the expectation based on our biochemical and structural results for individual RRM domains. Transcripts of two genes associated with neurodevelopment, *NTRK2* and *WNT3*, were recently reported as targets of RBM45. RBM45 was found to bind to the introns of these transcripts, introns 14 and 16 of *NTRK2* pre-mRNA, and intron 2 of *WNT3* pre-mRNA (5), all of which contain a large number of GAC and GACG motifs. These *in vivo* results support our hypothesis that RBM45 tends to bind RNAs containing multiple GAC or GACG motifs. However, because of the short sequence lengths, multiple GAC and GACG sequences are expected to present in most RNAs with thousands or more nucleotides, which is common in introns. Obviously, containing multiple GAC or GACG sequences is not enough to make it a target of RBM45. Our present and previous (14) biochemical and structural results showed that all RRMs of RBM45 bind RNA in extended conformation; therefore, whether an RNA molecule can be a target of RBM45 depends not only on its sequence but also on its secondary structure. As the shared recognition sequence of the three RRM domains of RBM45, GAC matches the sequence feature of the most common m^6^A modification site "DRACH" or "RRACH" motif (40, 41, 42, 43, 44), coupled with the fact that m6A-modified RNA tends to adopt single-stranded conformation (23), no matter whether or not the C-terminal RRM domain of RBM45 is a real m^6^A reader, RBM45 would likely prefer to bind RNAs containing the m^6^A modification, as demonstrated in a recently published work (5). In this manner, RBM45 could potentially mediate the regulation of RNA splicing and/or post-splicing events by m^6^A modification.

## Experimental procedures

### Cloning, protein expression, and purification

The DNA encoding the RRM3 domain of human RBM45 (RBM45^RRM3^, residues 370–476) was obtained using the Polymerase Chain Reaction (PCR) method from a codon-optimized full-length RBM45 gene synthesized by Sangon Biotech (Shanghai China) and cloned into pET-22b (+) vector with a C-terminal His-tag using restriction enzymes *Nde*I and *Xho*I. The recombinant plasmid was transformed into *Escherichia*. *coli* BL21 (DE3) cells and culture in LB medium containing 50 ug/ml ampicillin at 37 °C until OD_600_ reaches 0.6 to 0.8. Protein expression was induced by adding 0.3 mM isopropyl-β-D-thiogalactoside (IPTG). Cells were further incubated at 16 °C for 20 h and then collected by centrifugation. For protein purification, cells were suspended in a lysis buffer containing 20 mM Tris HCl, pH7.5, and 200 mM NaCl, and lysed by sonication. After centrifuging at 12,000 rpm for 60 min, the supernatant was loaded to a Ni-Chelating SFF column (Cytiva) and eluted with lysis buffer containing 200 mM imidazole. The eluate was desalted and loaded to an SP SFF column (Cytiva) equilibrated with a buffer containing 20 mM Tris HCl, pH7.5, and 50 mM NaCl, and eluted by a NaCl gradient. The purified protein was concentrated by centrifugal ultrafiltration for crystallization and biochemical experiments. The mutants of RBM45^RRM3^ were expressed and purified by the same method using plasmids generated by site-directed mutagenesis PCR. The primers used for molecular cloning are listed in Table S1.

### RNA and DNA oligonucleotides

The 5′-FAM labeled RNA and single-stranded DNA (ssDNA) used for fluorescence polarization assays and the ssDNA used for co-crystallization were purchased from General Biosystems.

### Crystallization

Crystallizations of RBM45^RRM3^ and RBM45^RRM3^–ssDNA complex were performed by sitting-drop vapor diffusing method. For the crystallization of RBM45^RRM3^, the protein was concentrated to 16.8 mg/ml in a buffer containing 20 mM Tris HCl, pH 7.5, and 180 mM NaCl. The beat crystals were obtained in a solution containing 20% (w/v) polyethylene glycol (PEG) 3350, 0.1 M Sodium citrate, pH 5.5, and 18% (v/v) 2-propanol at 16 °C. The crystal was soaked in a reservoir solution with 10% (v/v) glycerol added for several seconds and then flash-frozen in liquid nitrogen before data collection. For co-crystallization with ssDNA, the protein was mixed with a 7-nt 5′-GACGCAG-3′ ssDNA at a molar ratio of 1:1.2 and incubated on ice for 30 min before crystallization. The best crystals were obtained in a solution containing 0.1 M Sodium succinate, pH 5.3, and 8% (w/v) PEG 2000 at 16 °C. The crystal was soaked in a reservoir solution with 20% (v/v) glycerol for several seconds and flash-frozen in liquid nitrogen before data collection.

### Data collection and structure determination

All the X-ray diffraction data were collected at beamline BL18U of the Shanghai Synchrotron Radiation Facility (SSRF) at a wavelength of 0.97915 Å, using a Pilatus 6M detector, and processed with the HKL3000 package (45). The crystals of RBM45^RRM3^ and RBM45^RRM3^–ssDNA belonged to the *C*222_1_ space group and *P*2_1_ space group, respectively. The structures were solved by molecular replacement method with PHASER (46) in the CCP4 suite (47), using a model predicted by AlphaFold2 (18) as the search model. The models were then refined using REFMAC5 (48) and rebuilt with COOT (49). Clear electron density of DNA was observed in the map of RBM45^RRM3^–ssDNA structure, allowing unambiguous DNA model building with COOT. The final models were refined with PHENIX (50). The statistics of data collection and structure refinement are shown in Table 1.

**Table 1:** X-ray data collection and structure refinement statistics

Structures	RBM45^RRM3^	RBM45^RRM3^‒ssDNA
Data collection		
Wavelength (Å)	0.97915	0.97915
Space group	*C*222_1_	*P*2_1_
Cell dimensions		
*a*, *b*, *c* (Å)	71.26, 74.60, 195.84	42.40, 83.77, 77.76
*α, β, γ* (°)	90, 90, 90	90, 96.87, 90
Resolution (Å)	50.00–2.40 (2.49–2.40)	50.00–1.65 (1.71–1.65)
*R*_merge_	0.148 (1.357)	0.121 (0.974)
*I*/σ*I*	16.9 (2.0)	12.4 (2.7)
CC_1/2_	0.996 (0.714)	0.998 (0.841)
Completeness (%)	100.0 (100.0)	99.8 (99.8)
Redundancy	9.7 (10.2)	5.0 (5.1)
Total/Unique reflections	200,735 (20,694)	327,484 (64,857)
Refinement		
Resolution (Å)	35.49–2.41	38.97–1.65
No. reflections	20,388	64,589
*R*_work_/*R*_free_	0.183/0.221	0.158/0.207
No. atoms		
Protein	3069	3235
DNA	/	450
Ligand	2	18
Water	219	636
*B*-factors (Å^2^)		
Protein	39.3	18.8
DNA	/	31.2
Ligand	58.4	57.2
Water	42.6	36.7
R.m.s. deviations		
Bond lengths (Å)	0.004	0.004
Bond angles (°)	0.694	0.741
MolProbity score	1.27	1.01
Ramachandran plot		
Favored	97.9	99.0
Allowed	2.1	1.0
Outliers	0	0

∗Values in parentheses are for the highest-resolution shell.

### Fluorescence polarization analysis

The fluorescence polarization (FP) assay was used to analyze the RNA/ssDNA-binding affinities of RBM45^RRM3^ and its mutants. The FAM-labeled RNA or ssDNA at a final concentration of 100 nM was incubated with increasing concentrations of protein in binding buffer containing 10 mM Tris-HCl, pH 7.5, 200 mM NaCl, 1 mM DTT, 1 mM EDTA, and 5% (v/v) glycerol in dark at 25 °C for 30 min. The 535 nm fluorescence polarizations were measured by the SpectraMax Paradigm Multi-Mode detection platform (Molecular Devices) with an excitation wavelength of 485 nm. Three parallel measurements were performed for each sample. The data were fitted to the one-site model using the GraphPad software.

## Data availability

The structures of RBM45^RRM3^ and RBM45^RRM3^–ssDNA have been deposited into the Protein Data Bank (PDB) with accession numbers 8WQ3 and 8WQ5, respectively.

## Supporting information

This article contains supporting information.

## Conflict of interest

The authors declare that they have no conflicts of interest with the contents of this article.
