# Supplementary material for: Structural basis for RNA recognition by the C-terminal RRM domain of human RBM45
Source: J Biol Chem. 2024 Aug 8;300(9):107640. doi: 10.1016/j.jbc.2024.107640 (PMC11402289; doi:10.1016/j.jbc.2024.107640)
Supplement: Supplementary data [file mmc1.pdf]

Supporting information

Structural basis for RNA recognition by the C-terminal RRM domain of human RBM45

Xi Chen[1,2], Qinghao Wei[1,2], Zhongmei Yang[1,2], Xiaolei Chen[1,2], Shuoxuan Guo[1,2], Meiyu Jiang[1,2], Mingzhu Wang[1,2,3,*]

[1] Institutes of Physical Science and Information Technology, Anhui University, Hefei 230601, Anhui, China;

[2] School of Life Sciences, Anhui University, Hefei 230601, Anhui, China;

[3] Key Laboratory of Human Microenvironment and Precision Medicine of Anhui Higher Education Institutes, Anhui University, Hefei 230601, Anhui, China

* To whom correspondence should be addressed, wangmzh@ahu.edu.cn (MW)

Table S1. Primers for molecular cloning.

Mutation	Primers
Wild type	F: GGAATTCCATATGCAGATCCAGACCGAT<br>R: CCGCTCGAGGTAGGTACGCTGACG
R393Q	F: CCGGTTAAAGAAGCGCTGTTCATCGTTTTC<br>R: GAAAACGATGAACAGCGCTTCTTTAACCGGGGT
F395A	F: AAAGAACGTCTGGCGATCGTTTTCAAC<br>R: GTTGAAAACGATCGCCAGACGTTCTTT
E420A	F: GGTAACCTGATCGCGGTTTACCTGGTT<br>R: AACCAGGTAAACCGCGATCAGGTTACC
Y422A	F: CTGATCGAAGTTGCGCTGGTTAGCGGTAAA<br>R: ACCGCTAACCAGCGCAACTTCGATCAGGTT
Y431A	F: GGTAAAAACGTTGGCGCGGCGAAATACGCAGAC<br>R: TGCGTATTTCGCCGCGCCAACGTTTTT
Y431F	F: GGTAAA AACGTTGGCTTCGCGAAATACGCAGAC<br>R: GTCTGCGTATTTCGCGAAGCCAACGTTTTTACC
K433A	F: AACGTTGGCTACGCG GCGTACGC GACCGCATC<br>R: GATGCGGTCTGCGTACGCCGCGTAGCCAACGTT
K458A	F: AACGGCGTTCGTCTGGCGGTGATGCTGGCGGAC<br>R: GTCCGCCAGCATCACCGCCAGACGAACGCCGTT

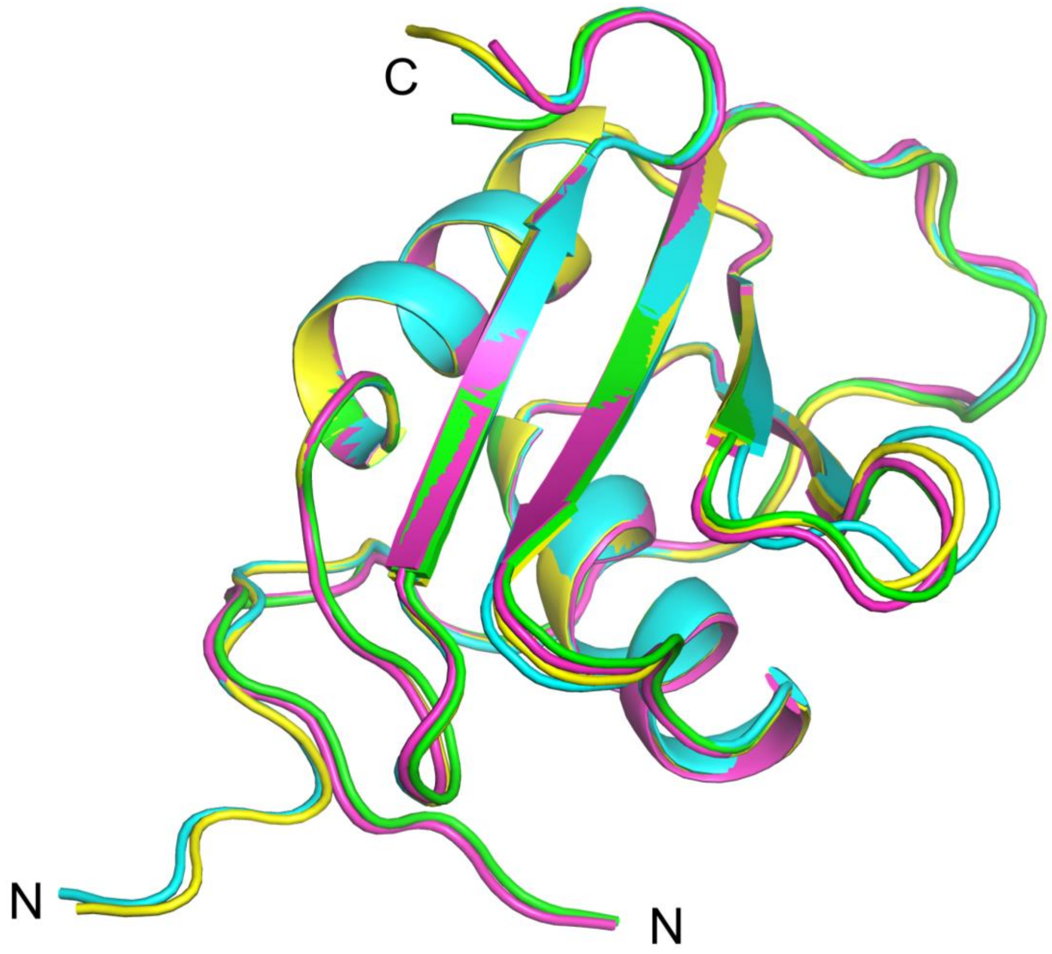

Figure S1. Superimposition of four RBM45^{RRM3} chains in the asymmetry unit.

Four chains are presented as green, cyan, magenta, and yellow cartoons, respectively.

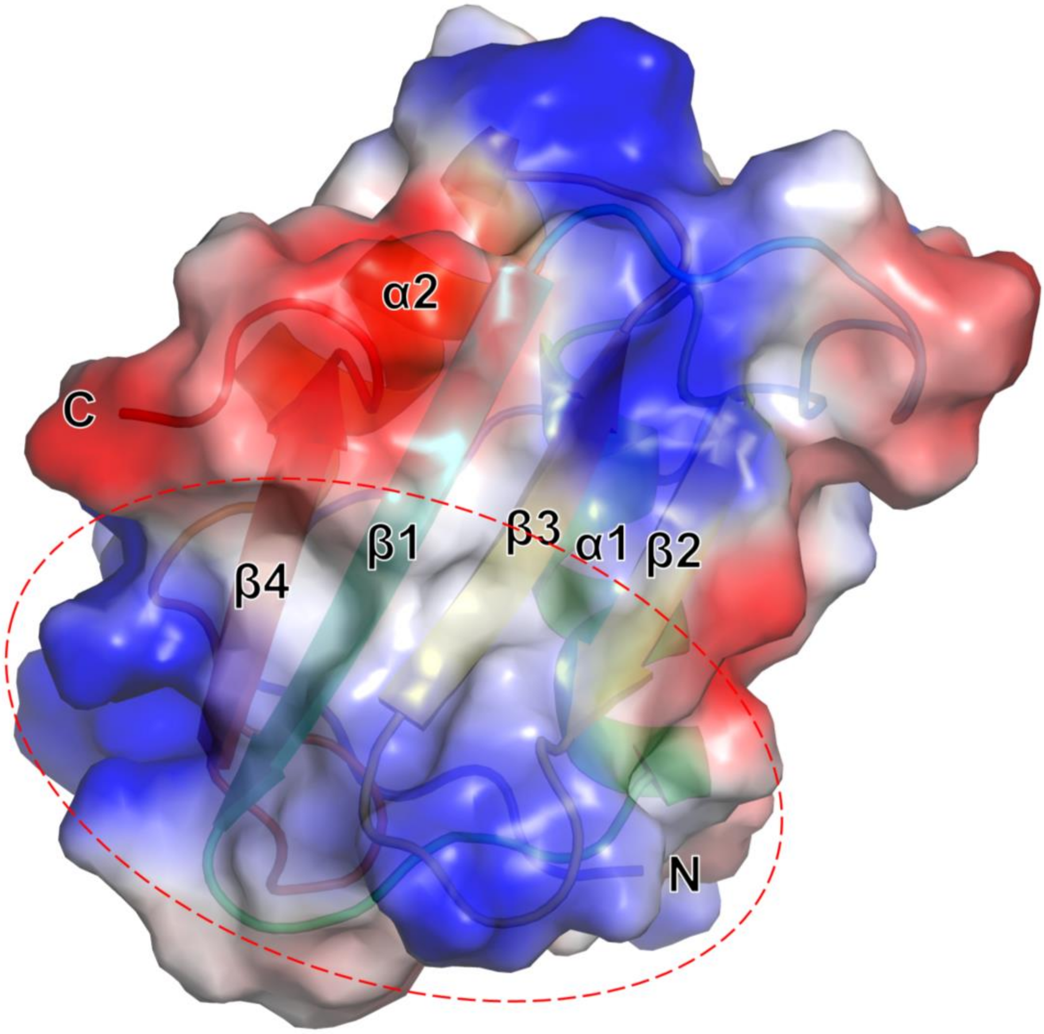

Figure S2. The electrostatic potential of the potential RNA binding surface of RBM45^{RRM3}. The potential RNA binding surface is indicated by a red dashed ellipse.

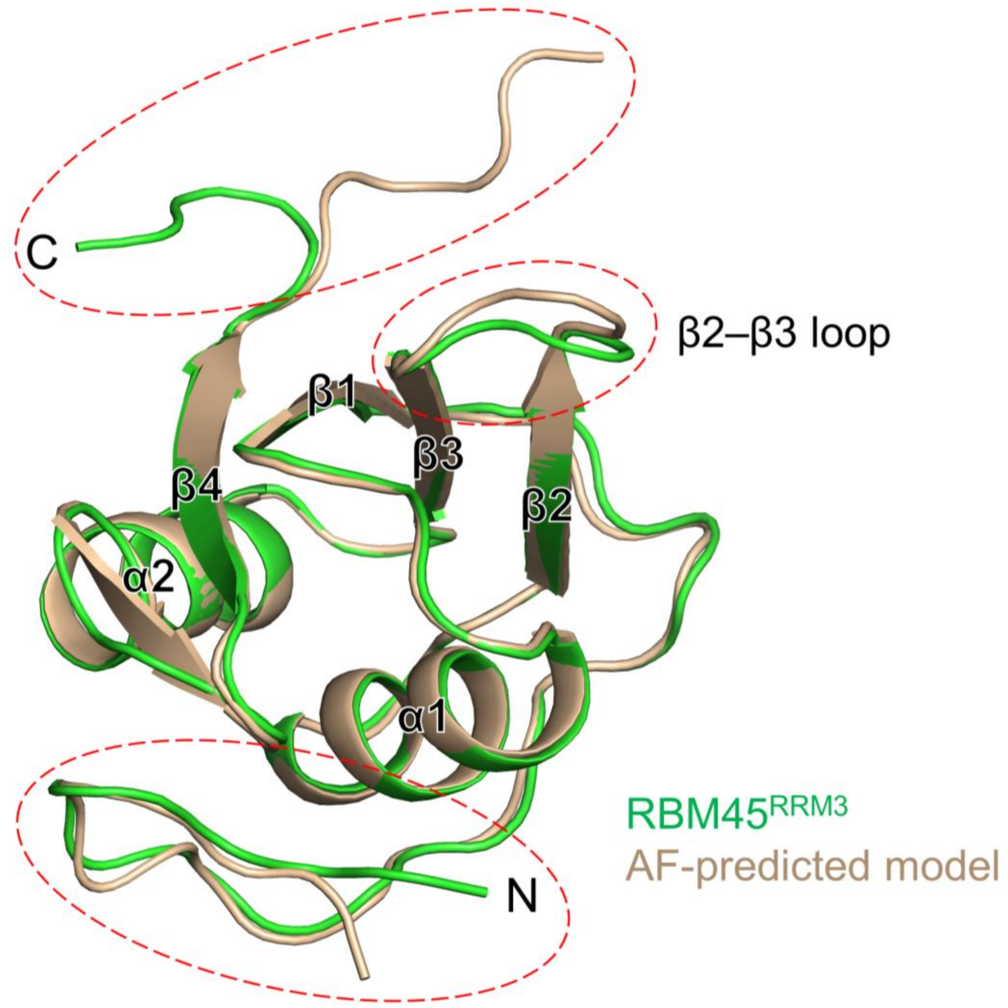

Figure S3. Superimposition of the crystal structure of RBM45^{RRM3} with the AlphaFold-predicted model. The crystal structure and AlphaFold-predicted model are presented as green and wheat cartoons, respectively. The N-terminus, C-terminus, and β2–β3 loop are enclosed by red dashed ellipses.

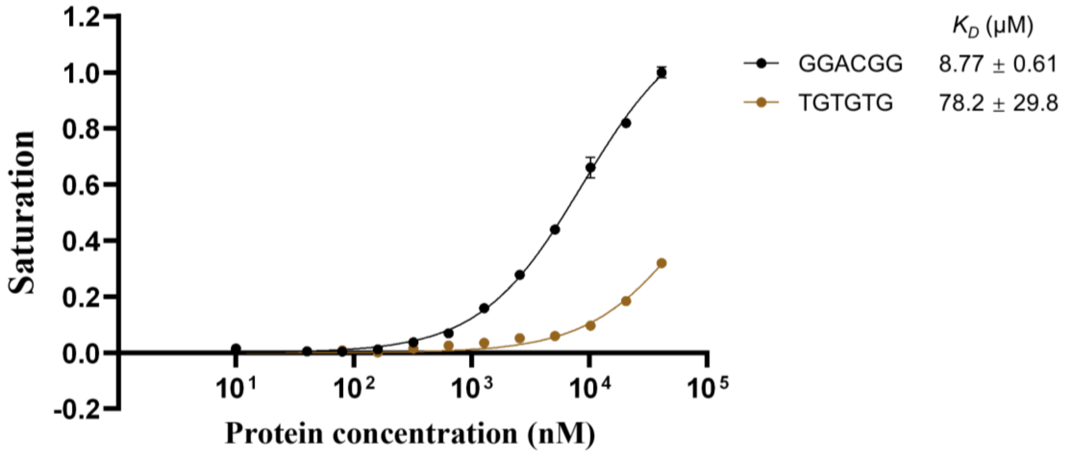

Figure S4. The FP assays for RBM45^{RRM3} binding to ssDNA. The sequences and K_Ds are shown on the right. The data shown here are the averages of three replicates. The error bars indicate the standard deviations of three replicates.

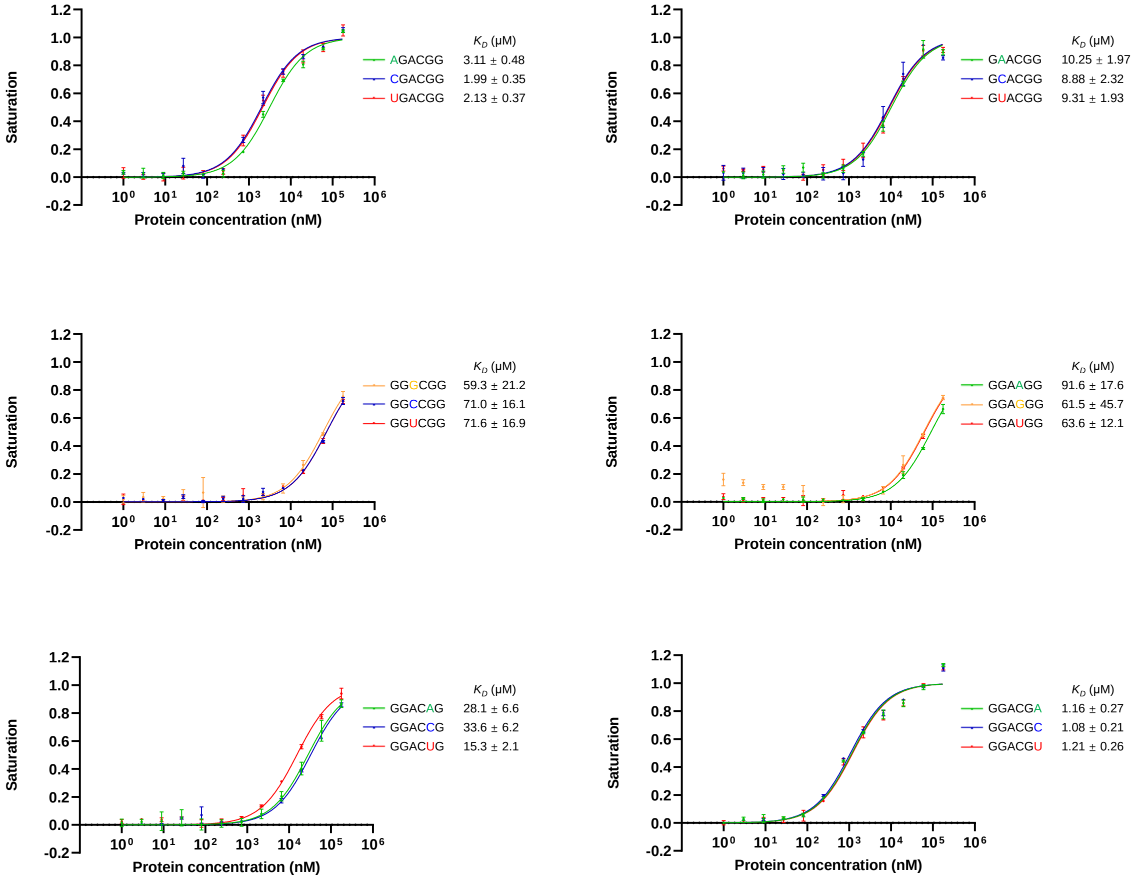

Figure S5. The FP assays for RBM45^{RRM3} binding to RNA with different sequences. The full results of Fig. 2B, including those have been shown in Fig. 2B, are shown here. The RNA sequences and K_Ds are shown on the right. The data shown here are the averages of three replicates. The error bars indicate the standard deviations of three replicates.

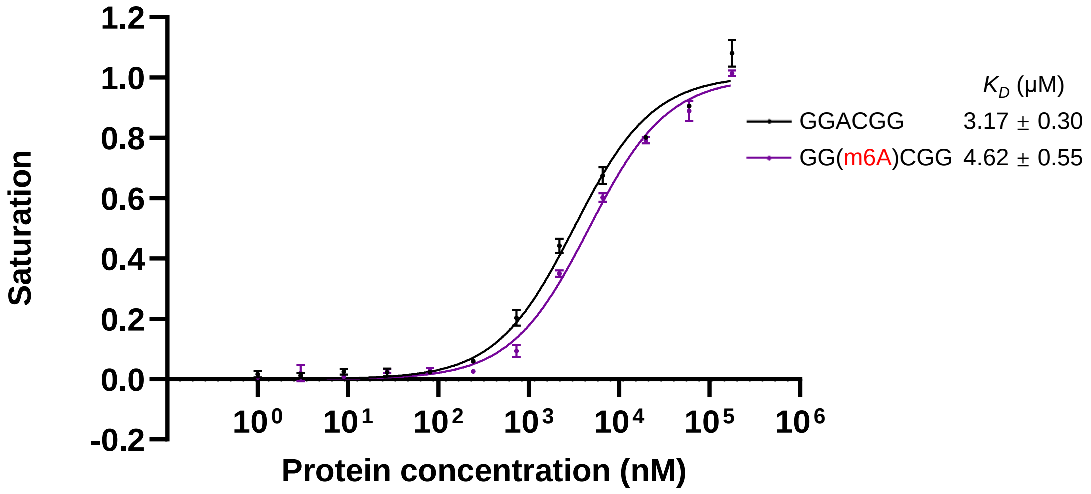

Figure S6. The m^{6}A modified RNA-binding affinity of RBM45^{RRM3}. The FP measurements of RBM45^{RRM3} binding GGACGG and GG(m^{6}A)CGG. The data shown here are the averages of three replicates. The error bars indicate the standard deviations of three replicates.

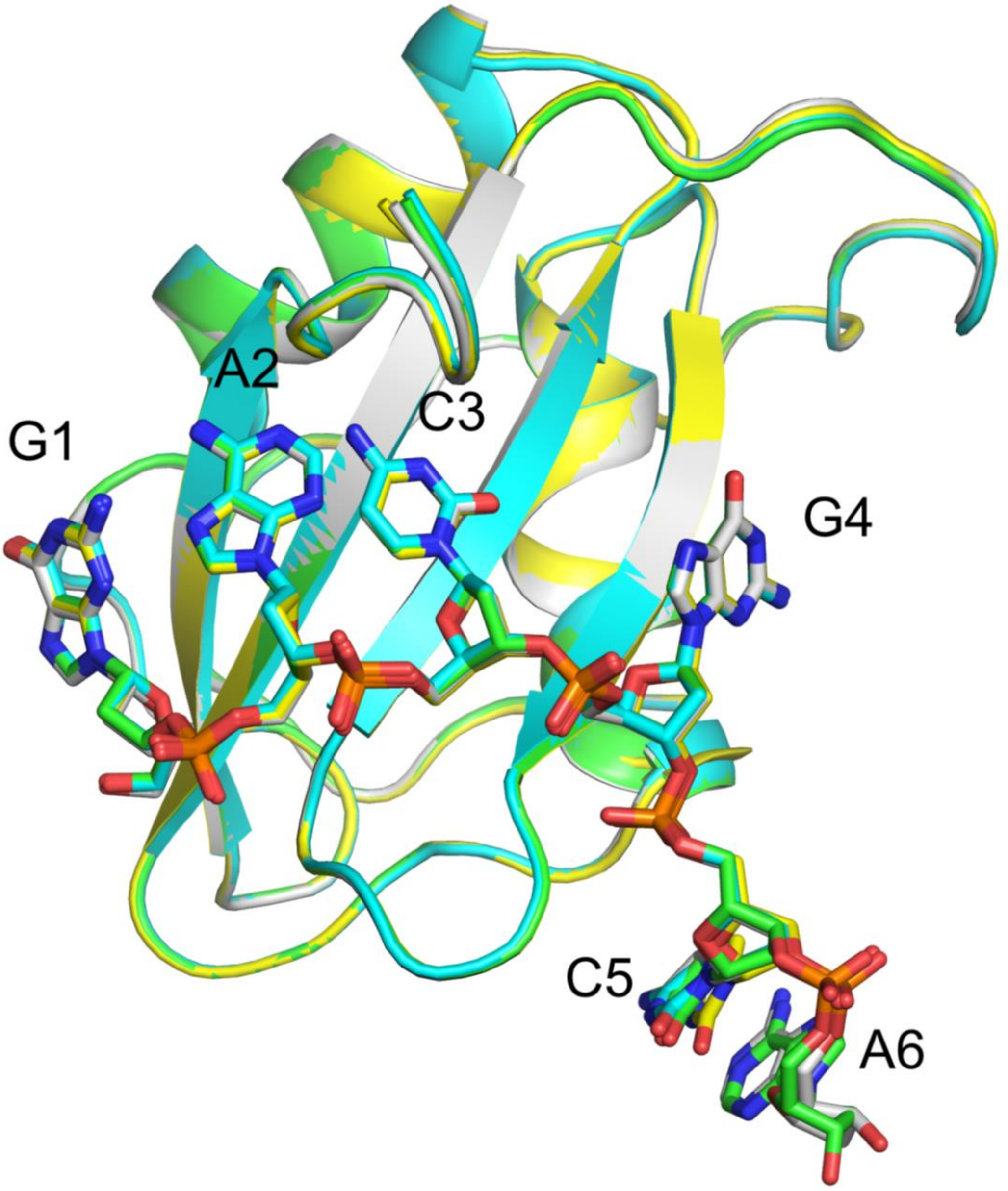

Figure S7. Superimposition of four RBM45^{RRM3} in the asymmetry unit and their bound ssDNAs. The four RBM45^{RRM3} chains are shown as green, cyan, gray, and yellow cartoons, respectively; their bound ssDNA are shown as green, cyan, gray, and yellow sticks, respectively.

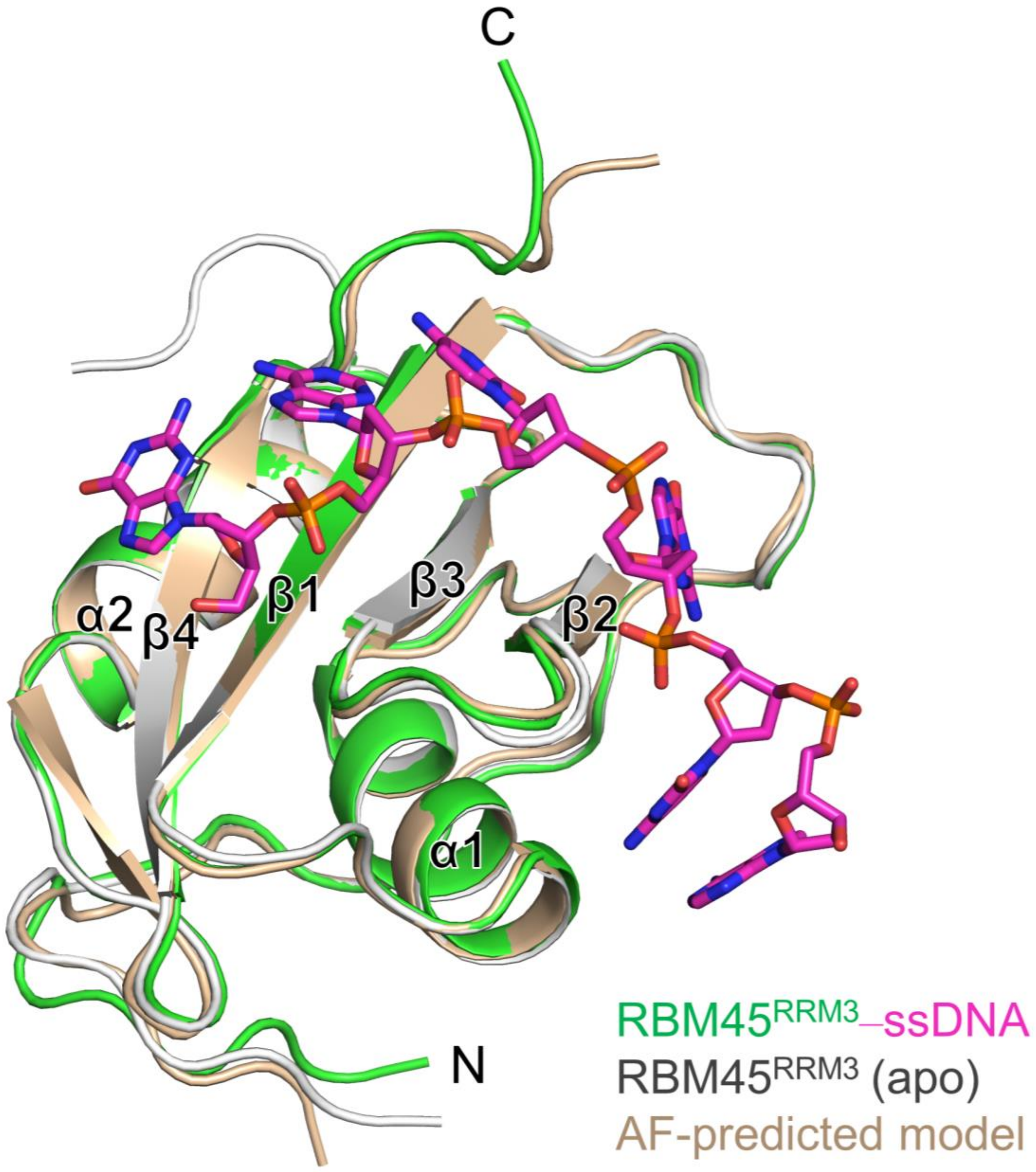

Figure S8. Superimposition of the complex structure, the protein-alone structure, and the AlphaFold-predicted model of RBM45^{RRM3}. RBM45^{RRM3} in the complex structure, the protein-alone structure, and the AlphaFold-predicted model are presented as green, gray, and wheat cartoons, respectively; the ssDNA bound to RBM45^{RRM3} is presented as magenta sticks.

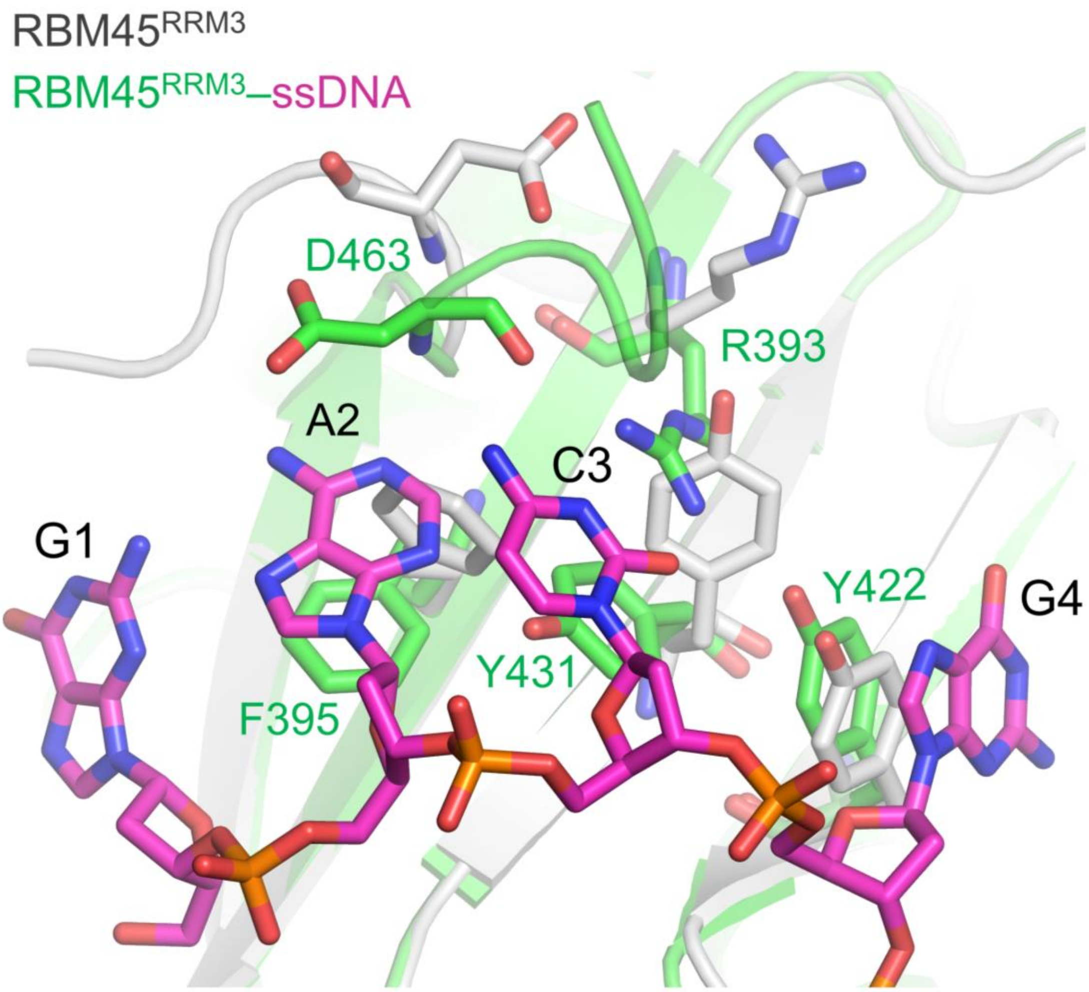

Figure S9. The different side-chain conformations of key RBM45^{RRM3} residues in the complex and protein-alone structures. The amino acid residues in complex structure and protein-alone structure are shown as green and gray sticks, respectively; the bound ssDNA is shown as magenta sticks.

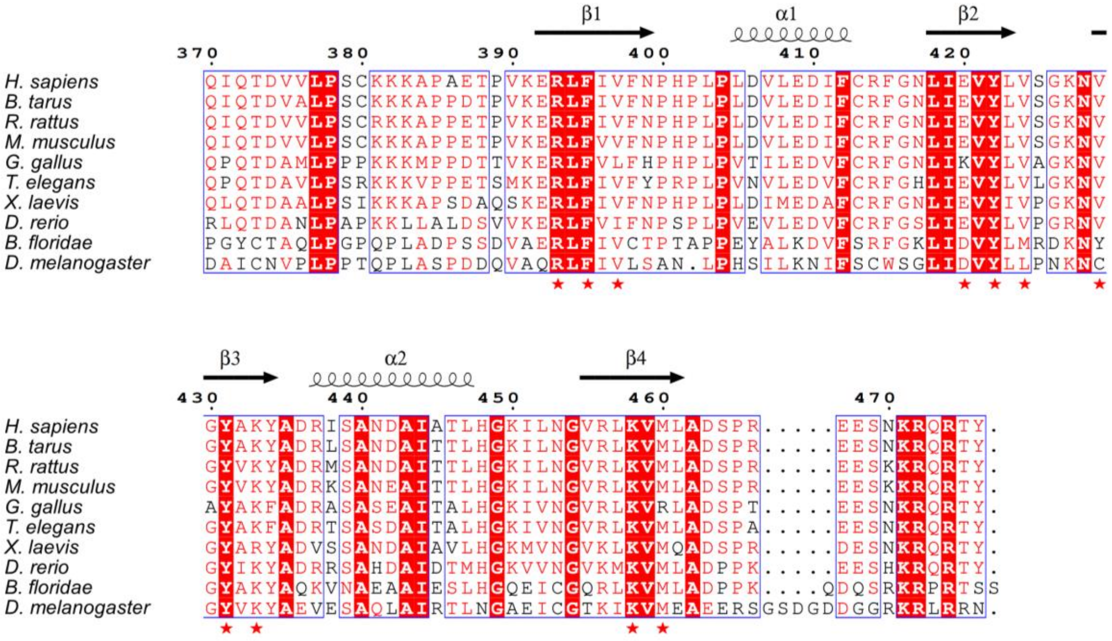

Figure S10. The cross-species sequence alignment of RBM45^{RRM3}. The amino acid sequence of human RBM45^{RRM3} is aligned with the corresponding sequences in RBM45s from *Bos taurus*, *Rattus rattus*, *Mus musculus*, *Gallus gallus*, *Trachemys scripta elegans*, *Xenopus laevis*, *Danio rerio*, *Branchiostoma floridae*, and *Drosophila melanogaster*. The identical residues are highlighted in red background; the similar residues are highlighted in red letters. Residues that interact with ssDNA through their side chains are indicated with red pentagrams.

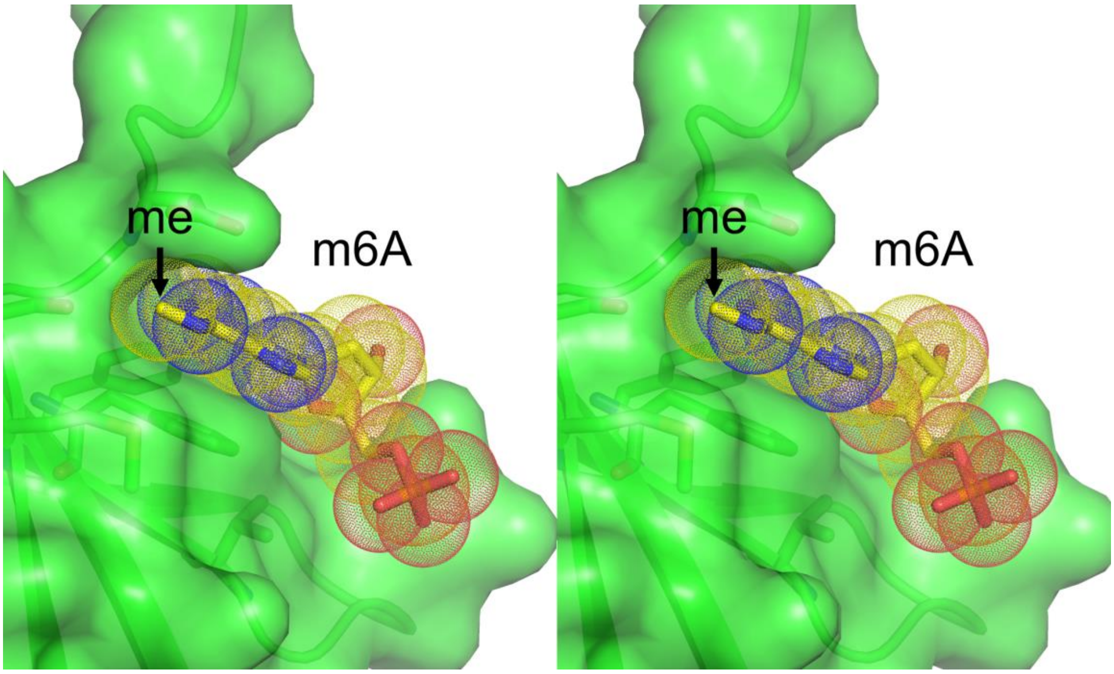

Figure S11. The stereoscopic view of the RBM45^{RRM3} binding a modeled m^{6}A. The surface of RBM45^{RRM3} is presented translucently in green. A modeled m6A in *syn* conformation is shown as yellow sticks with dots. The N6-methyl is indicated by a black arrow.
